# 

**Published:** 1906-10-15

**Authors:** 


### CONTENTS.

Event and Comment - - - - - - - - - 487

A Criticism—"Prophylaxis," - - By C. M. Wright, D.D.S. 496

Gold Inlays, - - - - - - By J. W. Lyons, D.D.S. 500

Mal-Occlusion of the Teeth and Its Intimate Relations to Carious Conditions, - - - - - By F. E. Williams, D.D.S. 513

With Our Editors - - - - - - - - - - 522

Indents - - - - - - - - - - - 535

Announcements - - - - - - - - - - - 538

